# Adiponectin and Atherosclerosis in Rheumatoid Arthritis

**DOI:** 10.1155/2014/358949

**Published:** 2014-06-04

**Authors:** Patrick H. Dessein, Linda Tsang, Ahmed Solomon, Angela J. Woodiwiss, Aletta M. E. Millen, Gavin R. Norton

**Affiliations:** ^1^Cardiovascular Pathophysiology and Genomics Research Unit, School of Physiology, Faculty of Health Sciences, University of the Witwatersrand, Johannesburg 2193, South Africa; ^2^Department of Rheumatology, Charlotte Maxeke Johannesburg Academic Hospital, Faculty of Health Sciences, University of the Witwatersrand, Johannesburg 2193, South Africa

## Abstract

In the present study, we examined the potential impact of adiponectin on carotid ultrasound determined atherosclerosis in 210 (119 black and 91 white) RA patients in mixed regression models. Total adiponectin concentrations were smaller in patients with compared to those without the metabolic syndrome (MetS) defined waist criterion (median (range) = 6.47 (1.23–34.54) versus 8.38 (0.82–85.30) ng/mL, *P* = 0.02, resp.); both total and high molecular weight (HMW) adiponectin concentrations were larger in patients with compared to those without joint deformities (7.97 (0.82–85.30) and 3.51 (0.01–35.40) versus 5.36 (1.29–19.49) and 2.34 (0.01–19.49) ng/mL, *P* = 0.003 and 0.02, resp.). Total and HMW adiponectin concentrations were associated with carotid artery plaque in patients with MetS waist (odds ratio (95% CI) = 0.87 (0.76–0.99) and 0.92 (0.85–0.99) per 1-standard deviation increment, *P* = 0.02 for both) and those without joint deformities (odds ratio (95% CI) = 0.94 (0.88–0.99) and 0.94 (0.89–0.99), *P* = 0.03 for both). Plaque prevalence was lower in patients without compared to those with joint deformities (23.4% versus 42.6, *P* = 0.004 in multivariable analysis). In RA patients with abdominal obesity or no clinically evident joint damage, adiponectin concentrations are reduced but nevertheless associated with decreased carotid atherosclerosis.

## 1. Introduction

Rheumatoid arthritis (RA) increases the risk of atherosclerotic cardiovascular disease (CVD) to a similar extent as diabetes [1–3]. Atherogenesis in RA remains poorly elucidated and current recommendations on CVD risk stratification [4] reportedly have major shortcomings [5, 6]. Hence, there is a need for identifying novel biomarkers of enhanced cardiovascular risk in RA [5, 6].

Adiponectin is an adipo(cyto)kine that was identified in 1999 [7]. Its production is paradoxically decreased in obesity [7, 8]. Numerous studies have documented that adiponectin exerts antidiabetic and vasculoprotective effects [8–14]. Amongst the different adiponectin isoforms, high-molecular weight adiponectin particularly protects against the development of diabetes [15] and CVD [16].

In cellular studies, adiponectin was shown to increase gene expression and protein synthesis of many proinflammatory and prodestructive molecules that participate in the pathophysiology of RA [17–19].

Both the production and effects of adipokines can be altered in patients with autoimmune diseases including RA [20, 21]. In this regard, whether adiponectin protects against CVD in RA is currently uncertain. Indeed, increased as well as reduced adiponectin concentrations in RA were reported by different groups [22–24]. Also both inverse or favorable and direct or paradoxically unfavorable associations of adiponectin concentrations with metabolic risk factors were found in RA [21, 24, 25]. With regard to direct effects of adiponectin on atherogenesis, we recently reported a cardiovascular risk factor independent direct relationship between adiponectin concentrations and early endothelial activation in white patients with RA [25]. Additionally, no association was found between adiponectin levels as well as carotid intima-media thickness (cIMT) and coronary artery calcification scores in RA [21, 26, 27]. However, compared to cIMT, carotid plaque is a more reliable indicator of severe atherosclerosis [28–34]. Thus, cIMT reflects mostly arterial media thickening in response to aging and elevated blood pressure whereas plaque represents intimal pathology and advanced atherosclerosis that links more closely to coronary heart disease risk factors and myocardial infarction [28–34].

In the present study, we examined the independent relationships of total and HMW adiponectin concentrations with cIMT and plaque. As the production and effects of adipokines on cardiovascular risk depend on pathophysiological context [21, 25, 35–39], we also determined whether the presence of conventional and nonconventional cardiovascular risk factors modified adiponectin concentrations and their associations with atherosclerosis.

## 2. Patients and Methods

### 2.1. Patients

The present study was conducted according to the principles outlined in the Helsinki declaration. The Human Research Ethics Committee (Medical) from the University of the Witwatersrand in Johannesburg, South Africa, approved the protocol (approval number: M06-07-33). Participants gave informed, written consent. This investigation forms part of an ongoing study on cardiovascular risk in RA [25, 36, 38, 39]. Two hundred and ten consecutive African patients (119 black and 91 white) that met the 1988 American College of Rheumatology and 2010 American College of Rheumatology/EULAR criteria for RA [40, 41] were enrolled. Carotid ultrasound was performed in all except 2 white patients. All invited participants agreed to participate. Data were missing in fewer than 5% of any of the recorded characteristics.

### 2.2. Assessments

Baseline characteristics and conventional metabolic risk factors were recorded using previously reported methods [25, 36, 38, 39]. Briefly, we recorded demographic features and lifestyle factors. Height, weight, and waist and hip circumference were measured using standard approaches. Overall adiposity, abdominal obesity, and fat distribution were estimated by the body mass index (BMI), waist circumference, and waist-hip ratio, respectively [28]. We recorded disease duration and rheumatoid factor status. Disease activity was assessed by the Clinical Disease Activity Index (CDAI) [42]. Extra-articular manifestations included the current or previously recorded (hospital record review) presence of pericarditis, pleuritis, Felty's syndrome, cutaneous vasculitis, neuropathy, scleritis or episcleritis, retinal vasculitis, glomerulonephritis, vasculitis affecting other organs, amyloidosis, keratoconjunctivitis sicca, xerostomia, Sjogren's syndrome, pulmonary fibrosis, bronchiolitis obliterans organizing pneumonia, cervical myelopathy, subcutaneous nodules, and rheumatoid nodules in other locations [43]. C-reactive protein concentrations were determined using immunoturbidimetric methods. Standard laboratory blood tests of erythrocyte sedimentation rate, renal and liver function, hematological parameters, lipids, and glucose were performed. The glomerular filtration rate was estimated using the Modification of Diet in Renal Disease equation [44]. Cardiovascular drug use was recorded.

Recorded metabolic risk factors included systolic, diastolic and mean blood pressure, lipid concentrations and ratios, and glucose levels. Hypertension was defined as an average systolic blood pressure ≥ 140 or/and diastolic blood pressure ≥ 90 mmHg or/and current use of antihypertensive medications. Dyslipidemia was diagnosed when the atherogenic index, that is, the cholesterol-HDL cholesterol ratio, was >4 [45]. Diabetes was identified as the use of glucose lowering agents or a fasting plasma glucose ≥ 7 mmol/L.

As reported previously, we also measured resistin concentrations [36].

BAS (see acknowledgement) and AS performed the carotid artery ultrasound measurements in private and public healthcare patients, respectively. Both operators obtained images of at least 1 cm length of the distal common carotid arteries for measurement of the intima-media thickness of the far wall from an optimal angle of incidence defined as the longitudinal angle of approach where both branches of the internal and external carotid artery are visualized simultaneously [46] and with high resolution B-mode ultrasound (Image Point, Hewlett Packard, Andover, MA, USA, and SonoCalc IMT, Sonosite Inc., Bothell, Wash, USA, used by BAS and AS, resp.) employing linear array 7.5 MHz probes. The details of the methodology used by BAS were reported previously [47]. The equipment used by AS involves the application of a unique semiautomated border detection program that was previously found to provide highly reproducible results [46]. The intima-media thicknesses in the left and right common carotid artery were measured and the cIMT was defined as the mean of of the two obtained values. Carotid artery plaque was defined as a focal structure that encroaches into the arterial lumen of at least 0.5 mm or 50% of the surrounding intima-media thickness value or demonstrates a thickness of >1.5 mm as measured from the media-adventitia interface to the intima-lumen interface [48]. Both operators were blinded to the cardiovascular risk profiles of the patients. Repeat ultrasound examinations by both operators on 23 patients revealed Spearman correlations between repeat cIMT measurements of 0.983 and 0.956 for BAS and AS, respectively, and the correlation between measurements made by BAS and AS was 0.926. Both operators identified carotid artery bulb or/and internal carotid artery plaque in 11 of these 23 patients with full agreement.

Total and HMW adiponectin concentrations were measured using solid-phase sandwich enzyme-linked immunosorbent assays (ELISA) (Quantikine HS, R&D Systems, Inc., Minneapolis, MN, USA). Their lower detection limits were 0.246 and 0.195 ng/mL, respectively. The inter- and intra-assay coefficients of variation were 6.5 and 3.5% for total and 8.5 and 3.0% for high molecular weight adiponectin, respectively.

### 2.3. Data Management Analysis

Dichotomous variables are expressed as proportions or percentages and continuous variables as mean (SD) or median (interquartile range (IQR)) when nonnormally distributed.

Associations of baseline characteristics with total and HMW adiponectin concentrations in the present cohort were previously reported and considered in multivariable analysis in the present study, that is, upon deciding which characteristics are potential confounders or mediators. These variables comprised age, sex, race, glomerular filtration rate, and waist circumference. We assessed the associations of HMW and total adiponectin concentrations with cIMT and plaque in Framingham score (calculated from age, sex, and major conventional risk factors), race, glomerular filtration rate, waist circumference, and C-reactive protein level adjusted mixed linear or logistic regression models as appropriate. The Framingham score was used rather than its individual components in order to avoid over-fitted models as these can produce false positive and false negative results.

Patients with RA that experience conventional risk factors or severe disease are reportedly at high risk of cardiovascular disease [1–6]. For these reasons together with the influence of physiological context on adipokine effects and our recent experience with adipokine metabolism in RA [21, 25, 35–39], we assessed the impact of patient characteristics on total and HMW adiponectin concentrations and their relations with carotid cIMT and plaque in subgroups with and without patient characteristics of interest in the present context. For this purpose, patients with a BMI of ≥30 kg/m^2^ and those that met the National Cholesterol Education Program for metabolic syndrome (MetS) waist criterion [49] were considered to sustain overall and abdominal obesity, respectively. When appropriate, patients were categorized in subgroups based on median values. In view of the small number of patients that were rheumatoid factor negative or had extra-articular features, sensitivity analysis in subgroups based on the presence or absence of these characteristics was not performed.

Statistical computations were made using the GB Stat program (Dynamic Microsystems, Inc., Silverspring, Maryland, USA) and SAS software, version 9.1 (The SAS Institute, Cary, NC). Significance was set at *P* value ≤0.05.

## 3. Results

### 3.1. Patient Characteristics

The demographic features, lifestyle factors, anthropometric measures, conventional metabolic risk factors, C-reactive protein concentrations, glomerular filtration rate, and use of antirheumatic and cardiovascular agents in the present cohort were previously reported [25]. The median (range) erythrocyte sedimentation rate was 14 (0–120) mm/hr.

Total and HMW adiponectin concentrations in all patients and relevant subgroups are given in Table 1. Total adiponectin concentrations were smaller in patients with compared to those without abdominal obesity and both total and HMW adiponectin concentrations larger in those with compared to those without deformed joints.

The mean (SD) cIMT and plaque prevalence in 208 of the patients is given in Table 2.

### 3.2. Independent Associations of Total and HMW Adiponectin Concentrations with Atherosclerosis

The relationships between total adiponectin and cIMT and plaque are shown in Table 3. Total adiponectin concentrations were not associated with atherosclerosis in all patients. However, total adiponectin concentrations were related to plaque prevalence in patients with but not without abdominal obesity and in those without but not with joint deformities.

The relationships between HMW adiponectin and cIMT and plaque are shown in Table 4. As applied to total adiponectin, HMW adiponectin concentrations were not associated with atherosclerosis in all patients and related to plaque prevalence in patients with but not without abdominal obesity and in those without but not with joint deformities.

The use of the Framingham score in our analysis may not have fully accounted for the confounding effect of age on atherosclerosis. However, in additional analysis in which we constructed models in groups as in Tables 4 and 5 but with adjustment for age, sex, race, and waist circumference as potential confounders, total and HMW adiponectin concentrations remained associated with plaque in patients with abdominal obesity and without joint deformities (*P* = 0.02 for each relationship).

In view of these findings we evaluated whether cIMT and plaque prevalence differed by abdominal obesity and joint deformity status. The respective results are given in Table 5. Plaque prevalence was numerically smaller in patients with compared to those without abdominal obesity and larger in patients with compared to those without joint deformities. The latter relationship was significant with an odds ratio (95% confidence interval) of 3.31 (1.46–7.51) for the association of deformed joint with plaque. In this regard also, obesity protects against joint damage in RA [50]. In the present study, the median (range) deformed joint count was smaller in patients with compared to those without abdominal obesity (5 (0–36) versus 10 (0–45), *P* = 0.05).

When, in additional analysis, we constructed models in groups as in Table 5 but with adjustment for age, sex, race, and waist circumference as potential confounders, the plaque prevalence remained higher in patients with compared to those without deformed joints (*P* = 0.01).

### 3.3. Independent Associations of Total and HMW Adiponectin Concentrations with Those of Resistin

In age, sex, race, glomerular filtration rate, cardiovascular drug use, and waist circumference adjusted models, total and HMW adiponectin concentrations were not significantly (*P* ≥ 0.2) associated with those of resistin in all patients and those with and without abdominal obesity or deformed joints. Also, upon further adjustment for resistin concentrations in Tables 3 and 4, total and HMW adiponectin concentrations remained associated with plaque (*P* = 0.02 to 0.002) in patients with abdominal obesity and without joint deformities.

Several recorded characteristics were nonnormally distributed in the present investigation. When we repeated the analyses using log transformed variables in the mixed regression models, the results were materially unaltered (data not shown).

## 4. Discussion

Previous studies on the potential involvement of adiponectin in enhanced CVD risk in RA have produced contradictory results [24–27]. Herein, we show for the first time that both total and HMW adiponectin concentrations are independently associated with reduced plaque prevalence in RA patients with abdominal obesity or clinical absent joint damage. A 1-standard deviation increment in total adiponectin concentration was independently associated with a 13 and 6% reduction in plaque prevalence amongst patients with abdominal obesity and clinical absent joint damage, respectively. Carotid artery plaque independently predicts incident cardiovascular event rates in non-RA subjects and patients with RA [51].

Our findings have at least 4 implications. First, as applies to osteoprotegerin [52] and retinol binding protein 4 (RBP4) [38], which is an adipokine that enhances atherogenesis, adiponectin is a promising cardiovascular risk biomarker that could improve cardiovascular risk stratification in RA [4–6]. Second, considering adiponectin concentrations only may be inadequate in estimating the impact of this adipokine in cardiovascular risk in RA. Indeed, despite the presence of reduced total and HMW adiponectin concentrations in patients with abdominal obesity or absent joint damage, it was in these groups that we observed inverse-adiponectin atherosclerosis relations. Thus, small rather large adiponectin concentrations concurred with a favorable association with cardiovascular risk in the present investigation. Taken together, increased adiponectin concentrations in RA do not necessarily translate into adiponectin mediated cardioprotective effects. Third, targeting adiponectin in an attempt to reduce RA severity [17–19] could be expected to additionally increase cardiovascular risk, at least in patients with abdominal obesity or less severe RA. Fourth, as previously documented by us [36–39], stratified analysis should be considered upon elucidating of the potential role of adipokines in CVD risk in RA.

Obesity is paradoxically associated with decreased incident cardiovascular event rates [53] and overall mortality in RA [54]. In this study, plaque prevalence was numerically reduced in patients with abdominal obesity. Congruently, we recently also found an independent inverse relation between abdominal adiposity and endothelial activation in RA [55]. The present investigation suggests that altered and favorable effects of adiponectin may constitute a mechanism that links abdominal obesity to reduced cardiovascular risk in RA.

Obesity also reportedly protects against joint damage in RA. In one study this relationship was explained by adiponectin concentrations [50]. In the present investigation, we also found an inverse association between abdominal obesity and joint damage. Joint deformity counts as well as radiographic damage in RA are associated with increased atherosclerosis [47]. Our current results further indicate that altered effects of adiponectin may additionally contribute to the link between mild RA and reduced cardiovascular risk.

We recently reported a paradoxically positive relation between adiponectin concentrations and endothelial activation amongst white patients with RA [25]. As also indicated by other investigators [56], we suggested that this finding represents a compensatory increase in adiponectin production aimed at reducing cardiovascular risk rather than a paradoxically altered adverse impact on atherogenesis [25]. Our current results support this possibility since total adiponectin concentrations exhibited a borderline inverse association with plaque prevalence in white patients (OR = 0.89 per 1-standard deviation increment, *P* = 0.07). Interestingly in this regard, we recently documented that RBP4 concentrations are paradoxically associated with reduced endothelial activation and, at the same time, also with enhanced atherosclerosis in RA [38].

High grade inflammation is associated with atherosclerosis and cardiovascular events in RA [57, 58]. In this regard, adiponectin is an anti-inflammatory adipokine [17–19, 21, 25] whereas resistin is proinflammatory [36, 59]. Indeed, resistin concentrations are associated with those of C-reactive protein in RA [36, 59]. Moreover, resistin levels are independently related to surrogate markers of early atherogenesis and may contribute to the link between inflammation and enhanced cardiovascular risk in RA [36]. Our analysis argues against circulating adiponectin and resistin being linked in RA and the adiponectin concentration-atherosclerosis relations were not explained by resistin levels.

In a recent investigation, Koskinen and colleagues [60] showed that in men with osteoarthritis (OA), circulating adiponectin concentrations correlate positively with levels of OA biomarkers and both circulating adiponectin levels and adiponectin concentrations released by cultured cartilage are associated with OA severity. Furthermore, when adiponectin was added at physiological concentrations to cultures of intact OA cartilage or primary OA chondrocytes, this adipokine enhanced the production of inflammatory and/or destructive factors nitric oxide, interleukin-6, and matrix metalloproteinases 1 and 3 in a mitogen-activated protein kinase-dependent manner. Hence, reported evidence together with the findings in the current study indicates that adiponectin can exert a dual role in arthritis comprising catabolic and proinflammatory effects on cartilage and protection against atherosclerosis.

We evaluated relationships of both total and HMW adiponectin concentrations with atherosclerosis in a relatively large and well characterized RA cohort. A substantial number of potential confounders or/and mediators were included in mixed regression models. Our study nevertheless has limitations. The cross-sectional design precludes drawing inferences on the direction of causality. We assessed joint damage by joint deformity counts rather than radiographic scores. However, we previously found that radiographic scores and joint deformities are strongly correlated in our setting (*R* = 0.808, *P* < 0.0001) [47]. We did not investigate matched healthy controls. Finally, circulating adiponectin concentrations may not necessarily represent its tissue levels.

In conclusion, the present study indicates that consideration of adiponectin can improve cardiovascular risk stratification amongst RA patients with abdominal obesity or mild disease. These findings merit further investigation in future longitudinal studies.

## Figures and Tables

**Table 1:** Total and high molecular weight adiponectin in all 210 RA patients and subgroups.

Groups	Number	Total adiponectin		HMW adiponectin	
		(ng/mL)		(ng/mL)	
		Median (IQR)	*P*	Median (IQR)	*P*
All	210	7.41 (4.89–11.97)	—	3.26 (1.27–5.74)	—
Population					
Black	119	7.41 (5.62–11.56)		2.65 (1.55–5.53)	
White	91	7.25 (5.31–12.83)	0.9	3.82 (2.10–6.00)	0.9
Age >55 years					
Yes	122	7.45 (5.20–13.58)		3.58 (1.86–6.18)	
No	88	6.84 (4.67–10.13)	0.2	2.67 (1.38–4.76)	0.1
≥2 major risk factors					
Yes	146	7.17 (4.62–11.39)		2.99 (1.53–5.40)	
No	55	8.48 (5.90–13.28)	0.1	3.78 (2.07–6.32)	1.0
Missing	9				
Obesity					
Yes	64	6.91 (4.32–9.76)		2.56 (1.26–4.96)	
No	140	7.58 (5.28–13.23)	0.7	3.47 (2.00–6.09)	0.9
Missing	6				
MetS waist					
Yes	100	**6.47 (4.32**–**10.01)**		2.66 (1.42–4.76)	
No	107	**8.38 (5.32**–**13.63)**	**0.02**	3.57 (2.03–6.48)	0.3
Missing	3				
RA duration >10 years					
Yes	109	7.41 (4.99–12.96)		3.44 (1.74–6.13)	
No	100	7.32 (4.32–11.29)	0.5	2.80 (1.53–4.94)	0.9
Missing	1				
CDAI >10					
Yes	93	7.51 (4.65–11.94)		3.33 (1.57–5.69)	
No	116	7.35 (5.04–12.83)	0.4	3.26 (1.98–5.87)	0.8
Missing	1				
ESR >12 mm/hr					
Yes	116	7.38 (4.50–11.29)		3.29 (1.69–5.60)	
No	87	7.87 (5.31–12.92)	0.4	3.26 (1.71–5.90)	0.8
Missing	7				
Deformed joints					
Yes	162	**7.97 (5.19**–**13.58)**		**3.51 (1.81**–**6.00)**	
No	47	**5.36 (3.55**–**8.56)**	**0.0003**	**2.34 (1.38**–**4.14)**	**0.02**
Missing	1				
RF positive					
Yes	161	7.41 (4.90–11.23)		3.27 (1.78–5.58)	
No	48	7.51 (3.95–15.03)	0.7	2.74 (1.29–6.49)	0.6
Missing	1				

Associations were identified in age, sex, race, glomerular filtration rate, cardiovascular drug use, and waist circumference adjusted models. Significant associations are shown in bold. RA: rheumatoid arthritis; HMW: high molecular weight; IQR: interquartile range; MetS: metabolic syndrome; CDAI: Clinical Disease Activity Index; ESR: erythrocyte sedimentation rate; RF: rheumatoid factor.

**Table 2:** Carotid atherosclerosis in 208 patients with rheumatoid arthritis.

Intima-media thickness, mm	0.710 (0.108)
Plaque	38.1

Continuous variable expressed as mean (SD) and categorical variable as proportion.

**Table 3:** Independent relationships of total adiponectin concentrations (1 SD increment) with carotid atherosclerosis in all RA patients and subgroups.

Groups	cIMT		Plaque	
	Partial *R*	*P*	OR (95% CI)	*P*
All	0.042	0.6	0.98 (0.91–1.05)	0.6
Population				
Black	−0.153	0.1	1.08 (0.94–1.30)	0.9
White	0.131	0.2	0.89 (0.77–1.01)	0.07
Age >55 years				
Yes	0.031	0.8	0.98 (0.89–1.07)	0.07
No	0.023	0.8	0.92 (0.75–1.02)	0.08
≥1 major risk factors				
Yes	−0.023	0.9	0.95 (0.89–1.01)	0.09
No	0.041	0.6	1.05 (0.90–1.02)	0.5
Obesity				
Yes	0.068	0.6	0.93 (0.84–1.02)	0.1
No	0.040	0.7	0.99 (0.90–1.09)	0.9
MetS waist				
Yes	0.112	0.3	**0.87 (0.76**–**0.99)**	**0.02**
No	0.003	1.0	1.08 (0.92–1.13)	0.7
RA duration >10 years				
Yes	0.129	0.2	0.94 (0.83–1.03)	0.2
No	−0.078	0.5	1.05 (0.94–1.15)	0.4
CDAI >10				
Yes	−0.017	0.9	0.92 (0.78–1.06)	0.2
No	0.090	0.4	0.99 (0.90–1.08)	0.7
ESR >12				
Yes	−0.072	0.5	0.96 (0.84–1.08)	0.4
No	0.129	0.2	0.97 (0.87–1.08)	0.6
Deformed joints				
Yes	0.068	0.4	1.00 (0.90–1.10)	0.9
No	−0.193	0.2	**0.94 (0.88**–**0.99)**	**0.03**
Rheumatoid factor positive				
Yes	0.023	0.8	0.99 (0.94–1.04)	0.9
No	0.091	0.6	0.91 (0.72–1.10)	0.3

Relationships were determined in Framingham score, race, glomerular filtration rate, waist circumference, and C-reactive protein concentrations adjusted models. Significant associations are shown in bold. SD: standard deviation; cIMT: carotid intima-media thickness; OR: odds ratio; CI: confidence interval; RA: rheumatoid arthritis; MetS: metabolic syndrome; CDAI: Clinical Disease Activity Index; ESR: erythrocyte sedimentation rate.

**Table 4:** Independent relationships of high molecular weight adiponectin concentrations (1 SD increment) with carotid atherosclerosis in all RA patients and subgroups.

Groups	cIMT		Plaque	
	Partial *R*	*P*	OR (95% CI)	*P*
All	−0.008	0.9	0.99 (0.96–1.02)	0.6
Population				
Black	−0.173	0.08	1.04 (0.94–1.33)	0.9
White	0.071	0.5	0.96 (0.95–1.01)	0.1
Age >55 years				
Yes	−0.057	0.6	0.99 (0.94–1.04)	0.07
No	0.008	0.9	0.95 (0.91–1.01)	0.1
≥1 major risk factors				
Yes	−0.042	0.8	0.96 (0.92–1.01)	0.07
No	−0.035	0.7	1.01 (0.95–1.12)	0.4
Obesity				
Yes	−0.008	1.0	0.96 (0.90–1.01)	0.1
No	−0.011	0.9	1.00 (0.96–1.05)	1.0
MetS waist				
Yes	−0.035	0.7	**0.92 (0.85**–**0.99)**	**0.02**
No	−0.006	1.0	1.02 (0.94–1.06)	0.6
RA duration >10 years				
Yes	0.059	0.6	0.97 (0.92–1.02)	0.2
No	−0.091	0.4	1.02 (0.97–1.07)	0.5
CDAI >10				
Yes	0.005	1.0	0.94 (0.86–1.02)	0.2
No	−0.002	1.0	1.00 (0.96–1.04)	0.9
ESR >12				
Yes	−0.013	0.9	0.96 (0.84–1.08)	0.3
No	0.038	0.7	0.99 (0.96–1.04)	0.7
Deformed joints				
Yes	0.018	0.8	1.00 (0.96–1.05)	0.8
No	−0.140	0.4	**0.94 (0.89**–**0.99)**	**0.03**
Rheumatoid factor positive				
Yes	0.008	0.9	1.00 (0.96–1.03)	0.8
No	−0.024	0.9	0.96 (0.86–1.05)	0.3

Relationships were determined in Framingham score, race, glomerular filtration rate, waist circumference, and C-reactive protein concentrations adjusted models. Significant associations are shown in bold. SD: standard deviation; cIMT: carotid intima-media thickness; OR: odds ratio; CI: confidence interval; RA: rheumatoid arthritis; MetS: metabolic syndrome; CDAI: Clinical Disease Activity Index; ESR: erythrocyte sedimentation rate.

**Table 5:** Carotid atherosclerosis in RA patients by abdominal obesity and clinical joint damage status.

Subgroups	Number	cIMT, mm	*P*	Plaque	*P*
MetS waist					
Yes	100	0.709 (0.098)		31.0	
No	107	0.713 (0.118)	0.9	45.8	0.5
Missing	3				
Deformed joint					
Yes	162	0.709 (0.104)		**42.6**	
No	47	0.719 (0.123)	0.4	**23.4**	**0.004**
Missing	1				

Continuous variables expressed as mean (SD) and categorical variables as proportions. Relationships were identified in Framingham score, race, C-reactive protein concentrations, and waist circumference adjusted models. Significant association is shown in bold. RA: rheumatoid arthritis; cIMT: carotid intima-media thickness; MetS: metabolic syndrome.
